# On the identity of the type species of the genus *Telema* (Araneae, Telemidae)

**DOI:** 10.3897/zookeys.251.3616

**Published:** 2012-12-18

**Authors:** Chunxia Wang, Carles Ribera, Shuqiang Li

**Affiliations:** 1 Institute of Zoology, Chinese Academy of Sciences, Beijing 100101, China; 2 Institut de Recerca de la Biodiversitat (IRBio) and Departament de Biologia Animal, Universitat de Barcelona, Avinguda Diagonal 645, 08028 Barcelona, Spain; 3 University of Chinese Academy of Sciences, Beijing 100049, China

**Keywords:** Taxonomy, diagnosis, haplogynae, cave, Eurasia

## Abstract

*Telema tenella* Simon, 1882, the type species of genus *Telema*, is the only species of family Telemidae reported from Europe and all other 39 congeners occur far from it.However, it has never been properly described. In this paper *Telema tenella* is redescribed and illustrated.

## Introduction

The genus *Telema* and its type species *Telema tenella* were described by Eugene Simon (1882) in the family Leptonetidae. The genus was later treated as a member of the subfamily Teleminae by Fage (1913), which was subsequently elevated to the status of family level, Telemidae Fage, 1913, by Petrunkevitch (1923). The family Telemidae now contains 8 genera and 61 species (Platnick 2012) from tropical Africa, East and Southeast Asia, and North and Central America. Its type genus, *Telema* Simon, 1882, is the largest genus in the family, with 40 species occurring in Guatemala, Southwest Europe, and East and Southeast Asia.

Prior to make global revision of the genus we decided to study the type species, which is insufficiently known, and to redescribe it details. This can allow us and other researches to conduct regional revisions of *Telema*. Although several authors provided descriptions of the type species *Telema tenella* (Simon 1893, Fage 1913, Yaginuma 1974, Ribera and Mateos 2000, Le Peru 2011), the morphological characters were not sufficiently detailed. Further, no information existed on the status of type material in Paris (Christine Rollard, personal communication). We made great efforts to find the species at the type locality, but failed. This suggested that *Telema tenella* has a restricted distribution. The species is known from six localities in Mount Canigó (Juberthie 1985) in the French Department of the Oriental Pyrenees. All localities were above 900 meters in elevation. More recently, this species has been recorded from la Cova del Far in the province of Girona, Spain, at 1100 meters in elevation (Ribera and Mateos 2000). However, Juberthie (personal communication) indicated that no specimens were recovered in five localities of Mount Canigó, and a recently conducted (2006–2007) survey in Cova del Far (Girona-Spain) also failed to detect specimens.

We had the chance to collect some specimens of Telemidae in a cave near the type locality of *Telema tenella* in 2011. The small cave was located next to Highway D115A, approximately 2000 meters from Banys de la Preste on the way to Prats of Molló, France (42°24.43'N, 2°24.37'E, elevation 1097 meters), which is approximately 9000 meters from the type locality. After comparing the specimen with original description, we treat them as *Telema tenella*, the type species of *Telema*. Herein, we describe these specimens.

## Materials and methods

Specimens were collected by direct searching, and subsequently were examined and measured using a Leica M205 C stereomicroscope. Further details were studied and measured with an Olympus BX41 compound microscope. The copulatory organs were examined and illustrated after they were dissected from the spiders’ bodies. Vulva were removed and treated in lactic acid before illustration. The right palp of male spider was illustrated. The specimens were preserved in an 80% ethanol solution. Photos were taken with an Olympus C7070 wide zoom digital camera (7.1 megapixels) mounted on an Olympus SZX12 stereomicroscope. The images were montaged using Helicon Focus image stacking software. All measurements were given in millimeters. Leg measurements were shown as: total length (femur, patella, tibia, metatarsus, tarsus). Leg segments were measured on their dorsal side.

The studied specimens were deposited in the Institute of Zoology, Chinese Academy of Sciences, Beijing (IZCAS).

## Taxonomy

### Family Telemidae Fage, 1913

#### Genus *Telema* Simon, 1882

##### Telema tenella

Simon, 1882

http://species-id.net/wiki/Telema_tenella

**Figure 1.**
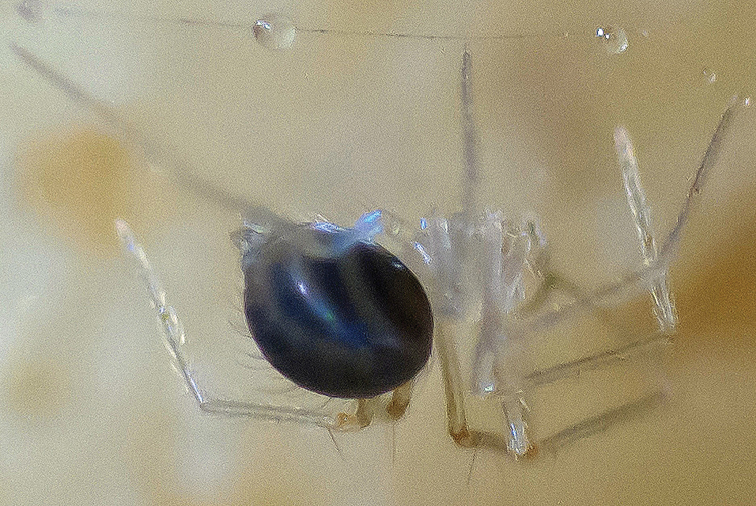
Subadultfemale hanging beneath web. Photo byEnric Planas in an unnamed cave in Banys de la Preste to Prats of Molló, France.

Figs 1 [Fig F2] [Fig F3] [Fig F4] –5

Telema tenella Simon 1882: 205 (♂♀); Simon 1893: 284, f. 240, 244 (♂); Fage 1913: 510, pl. 48–49, f. 1–21 (♂♀); Yaginuma 1974: 15, f. 1 (♂); Le Peru 2011: 142, f. 176 (♂♀).

###### Type material.

Not examined.

###### Material examined.

1 ♂ and 1 ♀ (IZCAS), unnamed cave, Banys de la Preste to Prats of Molló, France [42°24.43'N, 2°24.37'E, 1097 meters], Carles Ribera leg.

###### Diagnosis.

*Telema tenella* differs from other congeners by the distinct shape of male palp and female spermatheca. It is most similar to *Telema wunderlichi* Song & Zhu, 1994, a species from Hunan, China, but can be distinguished by the shape of carapace, five tiny denticles on the retromargin of chelicerae (four in *Telema wunderlichi*) and bent embolus (straight in *Telema wunderlichi*).

###### Description.

**Figure 2. F2:**
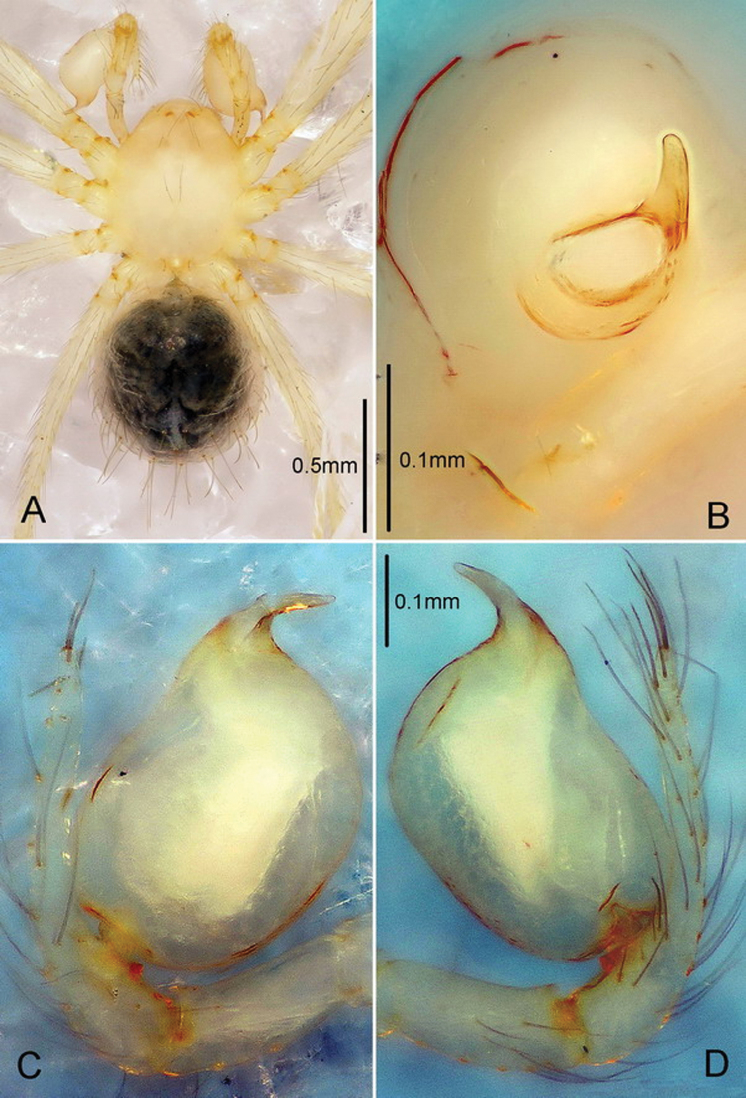
*Telema tenella*,male. Based on material from an unnamed cave in Banys de la Preste to Prats of Molló, France. **A** Habitus, dorsal view **B** Embolus tip, apical view **C** Right palp, retrolateral view **D** Right palp, prolateral view.

**Male** (Fig. 2A). Total length 1.40. Carapace 0.60 long, 0.55 wide. Abdomen 0.70 long, 0.64 wide. Sternum 0.40 long, 0.34 wide. Carapace, chelicerae, endites and labium yellow. Sternum and legs yellowish-white. Carapace with one pair of setae on clypeus, three medially and four on ocular area. Eyeless. Chelicerae (Fig. 4E) with one tooth and four tiny granulous denticles on the promargin, and five tiny granulous denticles on the retromargin of fang furrow. Anterior edge of labium with 4 strong and 8 small bristles. Patella I–IV dorsally with one long seta distally, tibia III IV with one long seta dorsally (position 0.5). Leg measurements: I 4.40 (1.33, 0.23, 1.32, 0.93, 0.59); II 3.91 (1.17, 0.23, 1.09, 0.86, 0.56); III 2.87 (0.87, 0.19, 0.75, 0.62, 0.44); IV 3.54 (1.09, 0.23, 0.94, 0.75, 0.53). Abdomen blue, covered with long setae. Male palp (Figs 2B–D, 4A–B, D). Bulb light yellow, pyriform, with embolus the only projection erected distally; embolus lamellar, curving, short, approximately 1/5 the length of bulb, and weakly sclerotized at the distal part.

**Figure 3. F3:**
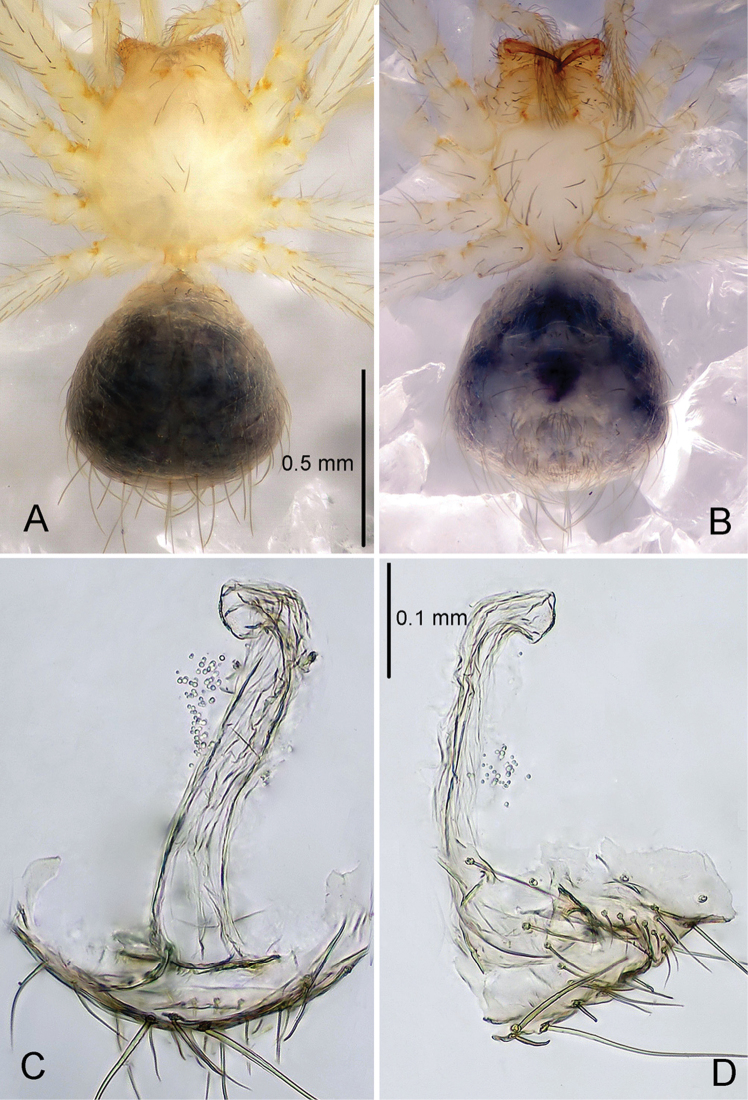
*Telema tenella*, female. Based on material from an unnamed cave in Banys de la Preste to Prats of Molló, France. **A**. Habitus, dorsal view **B** Habitus, ventral view **C** Copulatory organ, dorsal view **D** Copulatory organ, lateral view.

**Female** (Figs 3A–B). Similar to male in coloration and general features. Total length 1.30. Carapace 0.60 long, 0.55 wide. Abdomen 0.65 long, 0.65 wide. Sternum 0.40 long, 0.34 wide. Leg measurements: I 4.37 (1.33, 0.23, 1.32, 0.93, 0.56); II 3.92 (1.09, 0.23, 1.25, 0.81, 0.54); III 2.88 (0.71, 0.19, 0.86, 0.59, 0.53); IV 3.58 (0.94, 0.23, 1.09, 0.78, 0.54). Genital area (Fig. 5A) with a row of setae on epigynal area and a row of stout setae posterior to epigastric furrow. Spermatheca (Figs 3C–D, 5B–C) elongate, transparent, and tube-shaped, with the distal part curving ventrally.

###### Distribution.

In Pyrenees: France (Pyrénées-Orientales: caves Sainte-Marie and Can Britxot near La Preste; cave Sirach near Ria; caves Can Pey and La Fou near Arles-sur-Tech; copper mine near La Preste), Spain (Gerona, Cueva del Far).

###### Discussion.

The genus *Telema* contains 40 species distributed in Spain, France, Guatemala, Japan, Singapore, Borneo, Thailand, Vietnam, and China. Compared with the type species *Telema tenella*, some species in the genus have one prolateral apophysis on the cymbium; most species from China have a lager body size, more pigmented bodies, stronger embolus; and the species from Singapore, Borneo, and Thailand have different spermatheca in the female, either thin and strongly spiral or short and swelling. The taxonomic status of the species in *Telema* needs to be studied further as well as distributional limits of the genus.

**Figure 4. F4:**
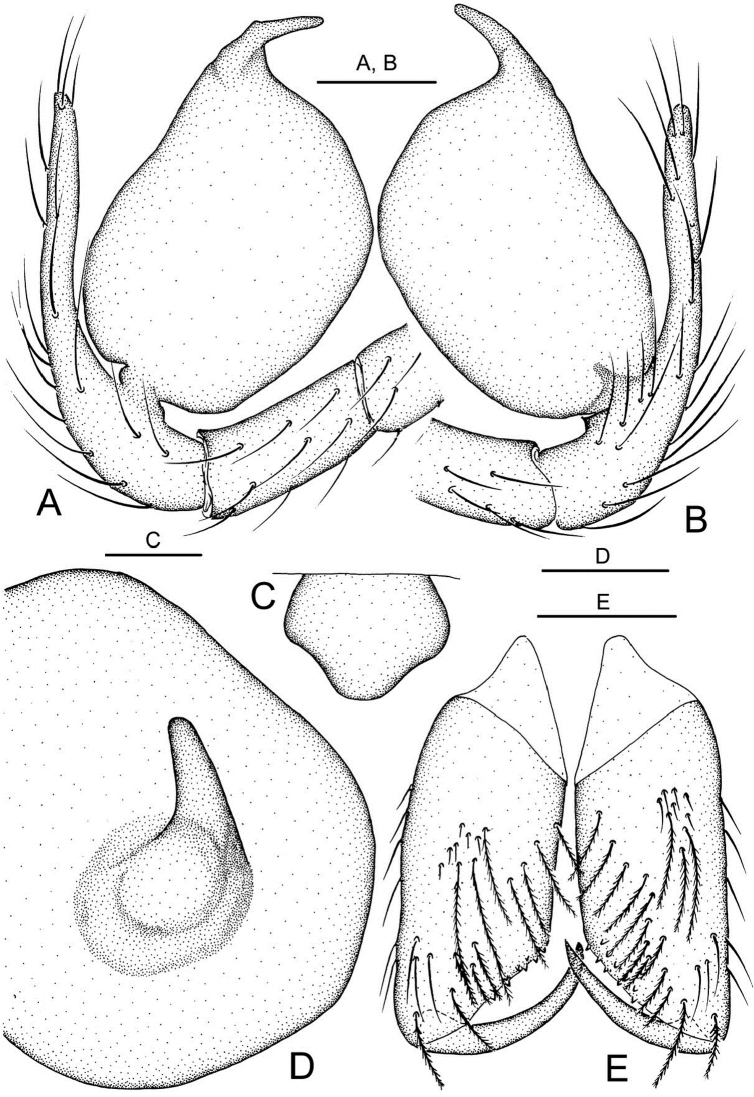
*Telema tenella*,male. Based on material from an unnamed cave in Banys de la Preste to Prats of Molló, France. **A** Right palp, retrolateral view B Right palp, prolateral view **C** Colulus, ventral view **D** Embolus tip, apical view **E** Chelicerae, posterior view. Scale bars: **A, B, E** 0.10 mm; **C, D** 0.05 mm.

**Figure 5.**
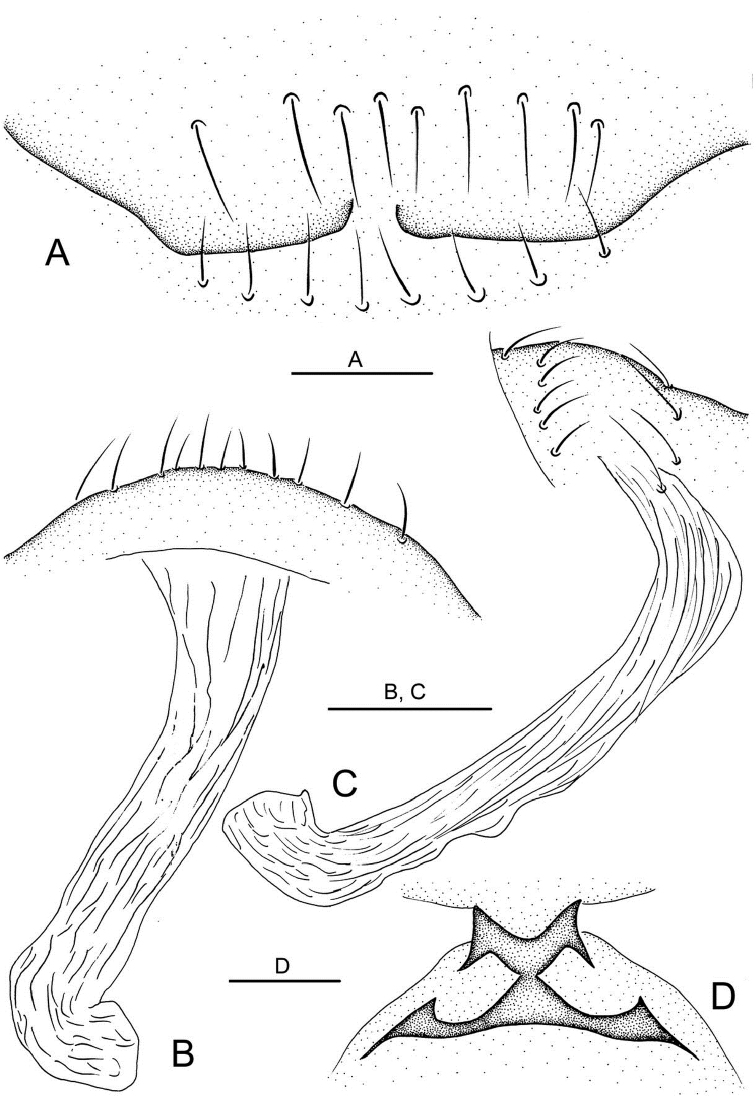
*Telema tenella*, female. Based on material from an unnamed cave in Banys de la Preste to Prats of Molló, France. **A** Genital area, ventral view **B** Copulatory organ, dorsal view **C** Copulatory organ, lateral view **D** Pedicel, dorsal view. Scale bars: **A**, 0.05 mm; **B, C, D** 0.10 mm.

## Supplementary Material

XML Treatment for Telema tenella
